# Effect of ice-lollies on the recovery time after anaesthesia: protocol for a cluster-randomised trial (Icesthesia)

**DOI:** 10.1186/s13741-026-00668-3

**Published:** 2026-03-19

**Authors:** Agnes S. Meidert, Roman Hornung, Anja Laska, Joseph Esser, Florian Brandes, Stephan Berthold, Melanie Borrmann, Volker Huge

**Affiliations:** 1 Department of Anaesthesiology, University Hospital LMU Munich, Marchioninistraße 15, 81377 Munich, Germany; 2 Institute for Medical Information Processing, Biometry and Epidemiology, University of Munich, Marchioninistraße 15, 81377 Munich, Germany; 3 Munich Center for Machine Learning (MCML), Munich, Germany; 4 Department of Critical Care Medicine and Anesthesiology, Schoen Clinic Bad Aibling, Bad Aibling, Germany

**Keywords:** Operating room performance, Post-anaesthesia care unit, Postoperative delirium, Postoperative nausea and vomiting, Postoperative pain, Recovery time; supportive care

## Abstract

**Background:**

Effective management in the post-anaesthesia care unit (PACU) is essential to ensure patient safety, comfort, and timely progression through the surgical care pathway. Many patients experience side effects such as nausea, vomiting, sore throat, hoarseness, dizziness, and disorientation. These symptoms can prolong recovery time. Ice-lollies are a pragmatic, non-pharmacologic intervention. We aim to test the hypothesis that offering ice-lollies in the PACU shorten the patients' length of stay in post-anaesthesia care.

**Methods:**

Icesthesia is a prospective monocentric cluster-randomised single-blinded controlled clinical superiority trial in 3140 patients admitted to the PACU of a large university medical centre after anaesthesia. Days are randomised to intervention days with ice-lollies plus standard of care and control days with standard of care only. Anonymised patient data is collected. The primary outcome is the length of time spent in the PACU until decision to discharge. Secondary outcomes are pain medication consumption, nausea and vomiting, and postoperative delirium at discharge.

**Discussion:**

This trial determines if offering ice-lollies to patients postoperatively reduces the length of stay in the PACU compared to standard of care.

**Trial registration:**

German Clinical Trials Register (DRKS00037179) on 11th June 2025.

**Supplementary Information:**

The online version contains supplementary material available at 10.1186/s13741-026-00668-3.

## Background

In the post-anaesthesia care unit (PACU), healthcare staff provide close observation and supportive care until patients regain stable vital signs and are sufficiently alert. Effective management in the PACU is essential to ensure patient safety, comfort, and timely progression through the surgical care pathway. After undergoing anaesthesia, many patients experience side effects such as nausea, vomiting, sore throat, hoarseness, dizziness, and disorientation. These symptoms can delay recovery and prolong the time needed before patients can safely return to their hospital ward or be discharged. Amongst the most common complaints after anaesthesia are sore throat or hoarseness. (Lehmann et al. 2010; Maruyama et al. 2004) Ice-lollies are a non-pharmacologic intervention in the PACU after surgery. They may have a beneficial effect on recovery: the temperature serves as cryotherapy, holding an ice lolly requires attention and keeps patients busy, ice lollies are often associated with positive childhood memories that can take away the focus from boredom, pain and nausea. There is limited evidence that this intervention reduces pain and emergence agitation in children after oral surgery (Sylvester et al. 2011) (Liang et al. 2023) However, if ice-lollies speed up recovery leading to faster discharge to the ward in a general surgical population is unclear. Therefore, we designed a prospective, single blinded monocentric trial investigating the effect of ice-lollies in the PACU on patients' length of stay. The hypothesis is that offering ice-lollies to postoperative patients leads to a faster recovery from the side effects of anaesthesia, thereby reducing the length of stay in the PACU. Secondary outcomes are the effect on consumption of pain medication, nausea and vomiting, and postoperative delirium.

## Methods/design

### Trial design

The proposed trial will be conducted according to the ethical principles based on the Declaration of Helsinki (World Medical Association Declaration of Helsinki 2013) and the guidelines of the International Council for Harmonisation Good Clinical Practice. The trial was approved by the local ethics committee (Ethikkommission bei der Medizinischen Fakultät der LMU München, chairperson R. Huber) under the registration number #25–0350. The trial was registered in the German Clinical Trials Register (DRKS00037179) on June 11, 2025.

Icesthesia is a single centre cluster-randomised single-blinded (with blinding to the primary outcome) controlled clinical superiority trial in 3,140 patients admitted to the PACU after anaesthesia. The study takes place at the LMU University hospital in Munich, Germany. This protocol is reported in accordance with the SPIRIT (Standard Protocol Items: Recommendations for Interventional Trials) guidelines.

### Patients

We will include patients from the age of 2 treated in the 25-bed PACU after surgery on workdays in one of the following specialties: neurosurgery, orthopaedic surgery, trauma surgery or ear-nose-throat (ENT) surgery. Patients are excluded from the study if they are not permitted oral intake of fluids or food including those with swallowing difficulties or other contraindications to safe oral consumption, or if they are transferred to an intermediate or intensive care unit postoperatively. Both elective and emergent procedures are included.

### Protocol

As this is a cluster randomized design with anonymised data collection with a minimal-risk, non-pharmacologic intervention, the requirement for individual informed consent was waived by the ethics committee. The intervention consists of offering ice lollies (water-based frozen confectionery with sugar and orange flavour) in addition to routinely provided refreshments in the PACU. The ice lollies are a commercially available product (Capri®, Langnese) supplied through the hospital food service in compliance with frozen food storage requirements. All ice lollies are identical in flavour and composition. One ice lolly (55 ml) contains 54 kcal and 12 g of carbohydrates, all of which are sugars. This represents a minor modification of standard care and does not involve any medical procedures or relevant nutritional changes. Given the cluster-level implementation within routine clinical workflow, obtaining individual consent was considered impracticable.

Every morning, the study day is randomised via a web-based randomisation tool (studyrandomizer.com, Rotterdam, Netherlands).(Study Randomizer 2017) Study days are randomized in a 1:1 ratio using permuted blocks with variable block sizes ranging from 2 to 6. The researcher collecting the data is blinded to randomisation details (e.g., blocking). On intervention days, ice-lollies are offered to all eligible patients by the nursing staff in addition to standard of care refreshments at the same time point at which oral fluids are routinely offered, and always at nurse discretion. Patients must be sufficiently awake and able to safely hold and swallow oral intake. No formal recovery score threshold is applied. On control days there is standard of care without ice-lollies. Standard postoperative refreshments in our institution include water, coffee and biscuits. Nurses are notified of the randomisation result by personal communication and a large sign in the middle of the PACU. To ensure protocol adherence and prevent cross-contamination, the freezer is kept securely locked on control days when no ice-lollies are provided. Adherence to the intervention will be documented. On intervention days, it will be recorded whether an eligible patient was offered and consumed an ice lolly. Patients who decline the ice lolly despite being eligible will remain in the intention-to-treat analysis according to the randomized day. A per-protocol analysis will additionally be performed including only patients who received the allocated intervention as planned. Anonymised patient data is entered prospectively at the bedside by a dedicated study investigator directly into a password-protected electronic Case Report Form (eCRF) hosted on the secure intranet server. Access is restricted to the research team and all activities are logged. No direct personal identifiers (e.g., name, hospital number, case number) are recorded. K-anonymisation is ensured by generalization via binning (age, height, weight, procedure) in predefined ranges. Data completeness is monitored during entry with immediate correction of missing or implausible values. Data collection ends when the patient is discharged to the general ward without follow-up. Patients are not re-identifiable in the study database.

### Safety considerations

Patients with diabetes or impaired glucose tolerance are not excluded from participation. Blood glucose monitoring and management are part of routine postoperative care in the PACU, and clinically relevant deviations are treated according to institutional protocols. The carbohydrate content of the intervention is comparable to standard postoperative oral intake and does not introduce additional risk beyond usual care.

### Outcomes

#### Primary outcome

The primary outcome is the time spent in the PACU from admission after surgery to the decision to discharge by the attending nurses and physicians. Time of decision to discharge instead of time of discharge was chosen to avoid procedural delays of patient transfer to the general ward due to staff shortage. PACU discharge is based on a standardized and routinely documented clinical assessment. This includes evaluation of respiratory and circulatory stability, temperature, urine output, bleeding, nausea/vomiting, sensibility and motor function, as well as neurological status assessed using the Glasgow Coma Scale, the Richmond Agitation-Sedation Scale, and the NUDESC score. Patients are discharged to the ward once all parameters are within acceptable clinical limits according to institutional practice.

#### Secondary outcome

Three secondary outcomes have three main components: pain, nausea and vomiting, and delirium. Pain includes the visual analogue scale at admission and discharge, and consumption of pain medication such as opioids, NSAID, metamizole and paracetamol. Nausea and vomiting is documented as separate outcomes, in addition the administration of any antiemetic medication intra- or postoperatively is recorded. Delirium is defined as a Nursing Delirium Screening Scale (NUDESC) at discharge ≥ 2. The NUDESC is also the communicated primary outcome to the staff and study team involved in data collection.

#### Blinding

Due to the nature of the intervention, blinding of patients and nursing staff to allocation is not feasible. However, the staff responsible for discharge decisions are not informed that PACU length of stay is the primary outcome. They are informed that delirium incidence assessed by the NUDESC score is the primary study endpoint. Discharge decisions are made according to routine clinical practice. Data are collected at the bedside by a doctoral researcher and entered directly into the electronic case report form. The researcher is unaware of the primary outcome.

### Statistical considerations and methods

#### Sample size

The primary outcome is length of stay in the post-anesthesia care unit (PACU), measured in minutes. As this is an exploratory study, no prior interventional data were available to inform the expected effect size. From an organizational perspective, a reduction of 5% in PACU length of stay was defined as the minimal relevant effect. Given a daily throughput of approximately 35–40 patients and responsibility for 16 operating rooms and additional anaesthesia workplaces, even small reductions in average PACU length of stay may translate into a meaningful cumulative gain in PACU capacity and potentially reduce operating room delays. Assuming a mean PACU stay of 90 min with a standard deviation of 40 min in the study population, this corresponds to an absolute reduction of 4.5 min and a standardized mean difference (Cohen’s d) of 0.11. The assumed mean PACU length of stay was derived from institutional administrative data used for nursing staffing and capacity planning. The sample size calculation assumed a two-sided type I error rate of 0.05 and 80% power. Under individual randomization, detecting this effect using a two-sample t-test would require approximately 2,600 patients. Because the study uses cluster randomization by day to avoid contamination, the sample size was inflated to account for within-cluster correlation. Assuming an average of approximately 30 patients per cluster and an intracluster correlation coefficient (ICC) of 0.01, the required sample size increased to 3,140 patients. Recruitment will stop once 3,140 patients with complete datasets have been enrolled; the planned study period is six months.

#### Statistical analyses

After patient enrolment is finished, data will be analysed. There is no interim analysis planned due to the short study period. Patient characteristics and clinical data will be descriptively analysed as appropriate after visual normality check. The proportion of missing data is expected to be very low, as data are entered prospectively at the bedside by a dedicated study investigator. If missing data remain minimal, a complete-case analysis will be performed. If the proportion of missing data should exceed expectations, appropriate sensitivity analyses will be considered. The analysis will be conducted as Intention-to-Treat (ITT) in all randomized patients according to group assignment and Per-Protocol (PP) with all patients who received the assigned intervention as planned. All tests will be two-sided, with α = 0.05.

Baseline characteristics will be summarised descriptively by treatment group. Continuous variables will be reported as mean (standard deviation) or median (interquartile range), as appropriate. Categorical variables will be presented as counts and percentages. No formal statistical testing of baseline differences will be performed.

To account for the cluster-randomized design with randomization at the level of the day, all inferential analyses will be performed using mixed-effects regression models including a random intercept for cluster (day).

For the primary outcome (PACU length of stay), a linear mixed-effects model will be used. If substantial deviation from normality is observed, an appropriate generalized linear mixed-effects model (e.g., gamma distribution with log link) will be applied. Binary outcomes will be analysed using generalized linear mixed-effects models with logit link.

For medication dosage outcomes with a high proportion of zero values, a two-part modelling approach will be applied: first, a mixed-effects logistic regression model will analyse the probability of receiving any medication (yes/no); second, among patients receiving medication, dosage will be analysed using an appropriate mixed-effects model depending on distributional characteristics.

For the primary and secondary outcomes, age range, sex, ASA physical status, surgery type, and duration and type of anaesthesia will be included as covariates. For the secondary outcomes pain medication and nausea/vomiting, the duration of stay in the PACU is included as an additional covariate.

Prespecified subgroup analyses are performed based on age (≤ 12 years and ≥ 75 years), sex, ASA classification, type of anaesthesia and surgery type (neurosurgery, orthopaedic surgery, ENT, plastic surgery). Subgroup analyses are considered exploratory.

#### Data management

Anonymized patient data is entered while the patient is in the PACU. Data are stored on a local server. Access to the database is limited to the study team with individual passkey and logged. There is no data monitoring committee or interim analysis planned due to the low-risk intervention and the short study period.

#### Dissemination plan

The results of the study will be published in a peer-reviewed journal, regardless of the results. Presentation of the results at a national conference is planned. Study protocol, statistical analysis plan, and anonymized data are available upon reasonable request from the principal investigator.

## Discussion

In our trial, we test whether ice-lollies offered to patients in the PACU lead to shorter time spent in the PACU until decision to discharge to the general ward. We chose this supportive, cost-effective intervention because it is easily translated into other health care systems, does not require a physician and has presumably no relevant side effects.

We expect faster recovery on days with ice-lollies as intervention for several reasons. First, the cold serves as neuronal thermal sensory stimulus helping to wean off residual sedation. Second, patients focus on hand-mouth coordination for some time, leading to a distraction from negative feelings of boredom and pain. Third, ice-lollies are a relief to the common side effects dry mouth and bitter or metallic taste after anaesthesia. Last, ice-lollies are for many people associated with happy childhood memories, helping to relieve stress symptoms.

If our intervention proves effective in shortening the length of stay in the PACU, this may lead to a smoother and more efficient recovery process for patients after anaesthesia. A reduced stay has the potential to improve the overall patient experience by minimizing discomfort and time spent in a high-monitoring environment, while at the same time preserving safety standards. From a medical-economic perspective, even modest reductions in recovery times can translate into meaningful benefits, including improved operating room throughput, better availability of postoperative recovery beds, and decreased staff workload. Capacity of postoperative recovery is crucial for a well organised OR, since overflow in the PACU lead to delay of subsequent surgeries. (Weissman et al. 2019) Both, physical space and trained staff, are a prerequisite for admission to the PACU. For sample size calculation we conservatively estimated a time reduction of five minutes per patient due to the activating effect of offering ice-lollies. This translates to an additional hour of one nurse per day who can care for three patients, an effect that could ease blocking of OR due to PACU overflow. (Macario et al. 1999) In high-volume surgical settings, these effects may contribute to both enhanced quality of care and more efficient use of healthcare resources.

The effect of the intervention in our study on secondary outcomes has been shown in preliminary studies by other researchers. A study in 92 children undergoing tonsillectomy randomised to receive an ice-lolly after anaesthesia observed lower pain scores in the intervention group, but no difference in pain medication administration. (Sylvester et al. 2011) In our study, all age groups are included. To find out, whether the cold reduces pain in ENT-surgery patients only, suggesting a local anaesthetic effect, we analyse the effect on surgical subgroups separately. If ice-lollies show a beneficial effect over all ages and surgical procedures, the benefit of the intervention seems to be a central anti-nociceptive effect such as positive memories or distraction from negative feelings. Another secondary outcome is nausea and vomiting. Some team members may be concerned, that oral intake of 55 ml of frozen, orange-flavoured liquid may lead to an increased rate in nausea and vomiting. A recent meta-analysis showed a beneficial effect of early postoperative fluid intake on nausea without an increase in vomiting. (M R Kang et al 2025) However, usually only water is offered to patients, therefore the analysis of postoperative nausea and vomiting is important in our study to detect possible negative side effects. The third secondary outcome is the effect on postoperative delirium reflected by the routinely assessed NUDESC at discharge from the PACU. Another study demonstrated a vast reduction of emergence delirium in children after oral surgery when they received an ice-lolly.(Liang et al. 2023) In our study, delirium was communicated as primary outcome to the staff in the PACU and the investigators who collect the anonymized patient data. Thereby, we avoid bias towards the primary outcome (Hawthorne effect), which is a strength of our study. Since we also include orthopaedic surgery with many elderly and frail patients, the analysis of this outcome might offer valuable insight into a non-pharmacological prevention strategy of postoperative delirium in an adult patient group.

One limitation of our study is the single-centre design. However, due to the cluster randomization, a high number of patients is expected to be enrolled in a short period of time. Furthermore, several patients who are offered ice-lollies will decline the intervention, which is the reason for the per-protocol analysis. Patients declining the intervention reflect real-world practice rather than protocol deviation. It is possible, that the number of patients actually accepting the intervention compared to the number of patients enrolled on control days will be considerably smaller, which could be another limitation. However, regarding the secondary outcomes, this trial includes 30 times more patients than the largest of the previous trials investigating the effects of this intervention; therefore, a conclusion based on sound data is expected. The strengths of our study are the blinding of the staff towards the primary outcome, the large number of patients and the pragmatic nature of the intervention. This protocol can serve as a template for a feasible study design in perioperative medicine.

In summary, we will perform a cluster randomized trial to test the hypothesis whether offering ice-lollies in the recovery area leads to a shorter length of stay in postoperative anaesthesia care.

### Trial status

Patient recruitment began in July 2025 and is expected to be completed in February 2026. This article is based on the most recent version of the study protocol (V 1.0, 11th June 2025). Publication of the protocol after beginning of recruitment is due to blinding of the primary outcome.

## Supplementary Information

Supplementary Material 1.

## Data Availability

Study protocol, statistical analysis plan, and anonymized data are available upon reasonable request from the principal investigator.
